# Compact and low mutual coupling 4 × 4 wideband MIMO antenna design for 5G millimeter-wave applications

**DOI:** 10.1038/s41598-026-39770-3

**Published:** 2026-03-25

**Authors:** Mohamed Edries, Hesham A. Mohamed, Dalia N. Elsheakh, Sherif Hekal

**Affiliations:** 1 Department of Electronics and Communication, Higher Institute of Engineering, El-Shorouk City, Cairo, Egypt; 2 Microstrip Department, Electronics Research Institute, Nozha, 11843 Cairo Egypt; 3 Electrical Department, Faculty of Engineering Technology, Elsewedy University of Technology- SUT, Cairo, Egypt; 4 Electrical Engineering Department, Faculty of Engineering, Benha University, Shoubra, Cairo, Egypt

**Keywords:** Millimeter-wave (mm-Wave), Wide-bandwidth, Mutual coupling reduction, Fifth generation (5G), Multiple-input-multiple-output (MIMO), Engineering, Physics

## Abstract

This paper presents a wideband 4 × 4 Multiple-Input-Multiple-Output (MIMO) antenna system operating within the 5G millimeter-wave (mmWave) Frequency Range 2 (FR2). The design targets enhanced mobile broadband (eMBB) applications and features four orthogonally arranged Greek cross-shaped slot antenna (GCSA) elements fed by microstrip lines to effectively reduce mutual coupling and ensure high isolation. Circular edges on the radiating elements are introduced to enhance the impedance bandwidth, while a strategically placed quadrilateral slot in the ground plane optimizes the radiation characteristics. The antenna is fabricated on a Rogers RT/Duroid 4003C substrate (ε_r_ = 3.55, thickness = 0.8 mm), resulting in a compact 40 × 40 × 0.8 mm^3^ structure. The proposed MIMO system achieves a measured impedance bandwidth of approximately 8 GHz, spanning 24 to 32 GHz at |S_11_|≤ − 10 dB, with a peak gain of 6 dBi at 27.5 GHz. The antenna exhibits directional radiation patterns perpendicular to the MIMO plane, yielding mutual coupling levels of − 25 dB and − 20 dB at 25 GHz and 29 GHz, respectively. Additionally, the design demonstrates excellent MIMO performance with an Envelope Correlation Coefficient (ECC) < 0.001 and Diversity Gain (DG) ~ 10 dB across the 23.93–31.18 GHz operational band. A comprehensive evaluation of scattering parameters, surface currents, radiation patterns, specific absorption rate (SAR), and diversity metrics confirms the robustness of the proposed design. Prototype measurements closely match the simulated results, validating the antenna’s suitability for 5G millimeter-wave communication systems.

## Introduction

The number of dense mobile applications has significantly increased due to the current global rollout of the fifth-generation (5G) network. Therefore, it is anticipated that mobile usage will increase exponentially during the next ten years^1,2^. As a result, the wireless communication ecosystem is facing significant challenges due to the exponential rise in mobile usage. Future wireless communication networks will need a thousandfold increase in capacity to accommodate the anticipated spike in traffic. Human-to-human (H2H) communications were the main purpose of the first generations of mobile communication networks, and they have reportedly met the required data rate and delay requirements with exceptional success^3^.

**Fig. 1:**
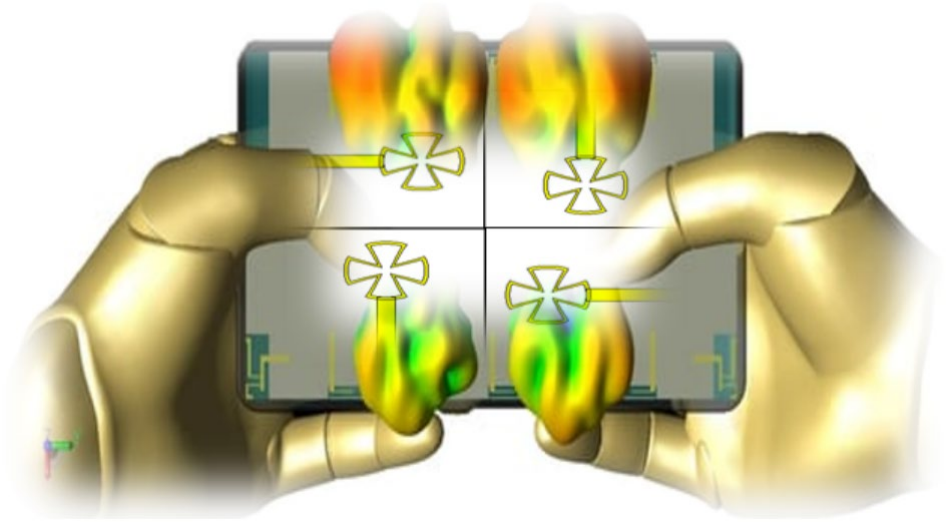
The proposed 4 × 4 MIMO system.

Figure 1 presents a three-dimensional view of the final proposed MIMO antenna configuration integrated within the mobile handset platform or portable devices and emerging technologies, such as autonomous vehicles, smart cities, and augmented reality, has intensified demand for high-speed, low-latency wireless connectivity, surpassing the capabilities of fourth generation (4G) networks^4–8^. The primary objective of this figure is to illustrate the overall antenna placement, orientation, and integration in the mobile environment, rather than the stepwise evolution of the antenna design. The detailed design procedure, including the intermediate development stages and performance refinement, is provided and discussed in the subsequent figures and corresponding sections for clarity and completeness. Since its global deployment in 2008, 4G has struggled with bandwidth scarcity and spectral inefficiency, prompting the need for advanced solutions^9^. The fifth generation (5G) network addresses these limitations by offering multi-gigabit data rates, ultra-reliable communication, and massive machine-type connectivity with minimal power consumption^10^. 5G aims to increase the data throughput, scalability, connectivity, and energy efficiency of mobile networks. One of the most sought-after subjects in telecoms research is 5G antenna design. User demands rise in tandem with technological advancements^11^.

The 5G uses millimeter-wave (mm-wave) frequencies for the unused spectrum. Moreover, the Federal Communications Commission (FCC) has allocated the 25–70 GHz millimeter-wave (mm-Wave) spectrum for 5G to harness these benefits, enabling unprecedented throughput and reduced interference^11–13^. The frequency bands of 27.5–29.5 GHz, 33.4–36.5 GHz, 37–40 GHz, 47–50.2 GHz, and 59.3–71.1 GHz have been categorized as belonging to the 5G mm-Wave spectrum by the International Telecommunications Union (ITU)^14^. Due to the minimal atmospheric absorption of electromagnetic waves in the 26, 28, and 33 GHz frequencies, 5G mobile communications are anticipated to function mostly in these regions^15^.

Globally, 5G deployment leverages two frequency ranges: FR1 (sub-6 GHz) for extended coverage and FR2 (mm-Wave, 24–100 GHz) for high-capacity short-range links. Notably, leading nations—including the United States, China, Japan, and the European Union—have adopted the 24.2–29.5 GHz band for 5G due to its worldwide availability, low atmospheric absorption, and minimal congestion from incumbent services^16^. For the deployment of 5G communication systems, intense research is presently being conducted on a few additional dual-band single-port antennas for millimeter-wave applications in addition to the work that has already been done. When compared to earlier communication network generations, these bands have demonstrated notable improvements and encouraging performance gains^15,17–19^. The integration of alternative techniques, such as multiple-input multiple-output (MIMO) systems, can address the difficulty of improving the quality of transmission in the high-frequency region.

Multiple-Input-Multiple-Output (MIMO) technology further enhances 5G performance by exploiting spatial multiplexing and diversity gains^19–34^. However, integrating multiple antennas into compact geometries introduces challenges, particularly mutual coupling between radiating elements, which degrades bandwidth and efficiency. Recent advances in metamaterials and orthogonal antenna arrangements have mitigated these issues, enabling high-isolation MIMO designs with compact footprints. Due to their extremely constrained space, mobile phones require designers to minimize antenna size without sacrificing performance.

The antenna community has several difficulties when it comes to mm-wave antenna design. The two main design goals for the practical use of printed antennas are bandwidth expansion and size reduction^20^. Low profile, dual or multiple bands of operation, low cost, minimal planar design, and squeezed size are all desirable characteristics for new RF designs’ antenna elements.

In addition to low correlation values between antennas, strong isolation, and a small distance between radiating parts can all help accomplish this improvement^21^. Significant coupling can occur in millimeter-wave systems due to the small distance between the antennas. The performance of the MIMO system could be adversely affected by this coupling^22^. The construction of 5G MIMO antennas operating at mm-wave without the need for decoupling techniques has been the subject of numerous recent studies^23–25^. As a result, the MIMO system’s layout is largely responsible for its mutual coupling. There is a coupling of − 20 dB at both frequencies, as seen in^23^. On the other hand^26,27^, show 20 dB isolation in the dual-band MIMO system. Furthermore, in^28^, an improved isolation of almost 20 dB is demonstrated. In a similar vein, the dual-band MIMO system exhibits 20 dB better separation. In^30^, A 4 × 4 MIMO antenna has an efficiency rate of 75%, an envelope correlation coefficient (ECC) below 0.001, an isolation level below − 20 dB, a peak gain of 1.83 dBi, and a reflection coefficient parameter of − 35 dB.

This paper presents a 4-port MIMO antenna array optimized for 5G mm-Wave applications extended from 24 to 32 GHz as shown in Fig. 1. The proposed design employs orthogonally configured Greek cross-shaped elements to suppress mutual coupling while circular edges and a quadrilateral ground plane slot enhance impedance bandwidth at |S11|≤ − 10 dB as 8 GHz and radiation efficiency about 80% over the operating band. Key achievements include a peak gain of 6 dBi at 28 GHz, inter-element isolation > 26 dB, and exceptional diversity performance envelop correlation coefficient (ECC) < 0.001, diversity gain (DG) > 10 dB. The proposed MIMO system is fabricated on a Rogers RT/Duroid 4003c substrate, with a 40 × 40 × 0.8 mm^3^ array. The measurement results are validated through simulation with Computer Simulation Technology (CST Microwave Studio) and measurements, demonstrating robustness for 5G integration.

The proposed antenna is a potential contender for the 5G antenna system due to the numerous significant qualities listed below.

- Simple design, quick fabrication, and planar geometry;
- Small size for integration into 5G devices;
- High isolation between the antenna parts without the use of a complicated decoupling structure;

The paper is organized as follows: the second section presents details of the single-element antenna design, Section three analyzes a 2-element MIMO subsystem, Section four evaluates the full 4 × 4 MIMO array’s scattering parameters, radiation patterns, and diversity metrics, and Section five presents the fabrication of the proposed MIMO System. While section six shows proposed MIMO performance parameters and section seven introduces the discussion of the results, and section eight concludes the paper.

### Design proposed antenna element

**Fig. 2:**
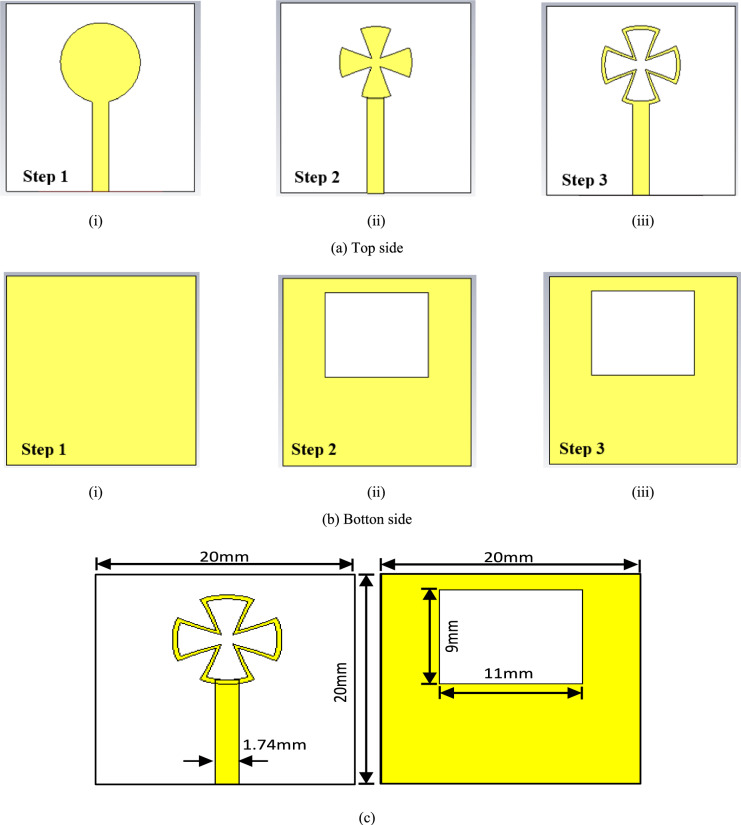

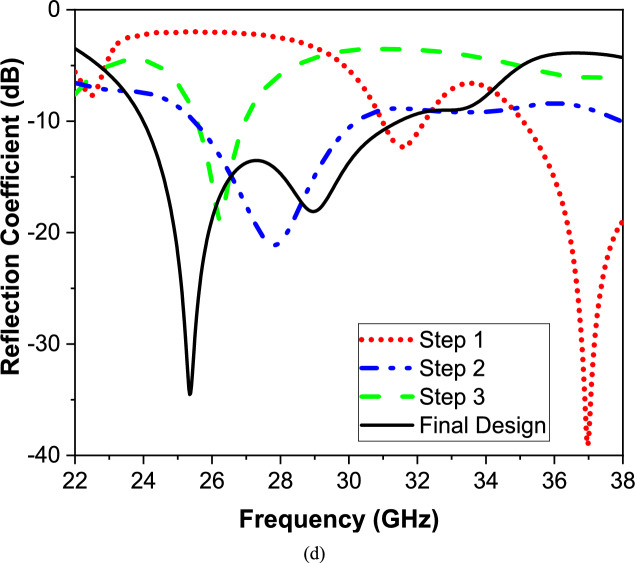
Design Steps (**a**) Radiation plane, (**b**) Ground plane, (**c**) Design model of a single antenna element, and (**d**) Reflection coefficient results.

Each MIMO antenna element’s construction is chosen to allow for resonant broadband operation. The first step in this technique is designing a composite structure with a circular patch. Figure 2 displays the single-unit configuration of the printed slot microstrip antenna. The proposed structure uses a 0.8 mm RO4003 substrate with ε_r_ = 3.55. The antenna has a total size of 40 × 40 mm^2^, which is suitable for mobile handheld devices. The antenna features a rectangular slot in its ground plane. In contrast, the 50 Ω microstrip line terminates with a small crescent on the opposite side of the substrate to enhance the matching impedance. The antenna is first constructed from a circular patch with radius Rp as the main radiator according to the following Eqs. (1) and (2)^34^:

1 $$R_{P} = \frac{F}{{\left\{ {1 + \frac{2h}{{\pi \varepsilon_{r} F}}\left[ {\ln \left( {\frac{\pi F}{{2h}}} \right) + 1.7726} \right]} \right\}^{1/2} }}$$

2 $$F=\frac{8.791\times {10}^{9}}{{f}_{r}\sqrt{{\varepsilon }_{r}}}$$

where *h* is the substrate thickness, *ε*_*r*_ is the relative dielectric constant *f*_*r*_ is the resonant frequency, and *F* is the Frequency-dependent term (defined in Eq. 2).

The preliminary design, as step one, is composed of a circular radiator with a complete ground plane, as shown in Fig. 2ia. The antenna operates around 28 GHz with a small bandwidth. Then, to increase the antenna bandwidth and reduce the resonant frequency, a rectangular slot is etched on the ground plane as shown in Fig. 2iib. The slot on the ground plane is used to increase the capacitance as well as increase the path length to be operated from 25 to 27 GHz. Furthermore, in step three, a Greek cross-shaped slot is etched on the radiator as shown in Fig. 2iiia. This slot shape and dimensions as shown in, added more capacitor value and enhanced the impedance matching. The proposed design enhanced the response at the lower frequency band; the circular radiator is changed to a crescent shape. The proposed design operates from 24 to 32 GHz, as illustrated in Fig. 2d. It should be mentioned that to modify the operating frequencies at these dimensions, comprehensive parametric investigations are conducted. The final dimensions of the proposed millimeter-wave antenna design are written on the configuration shown in Fig. 2c. The proposed structure has an operating band of 8 GHz expanded from 24 to 32 GHz with a reflection coefficient |S_11_|≤ − 10 dB as illustrated in Fig. 2d.

**Fig. 3:**
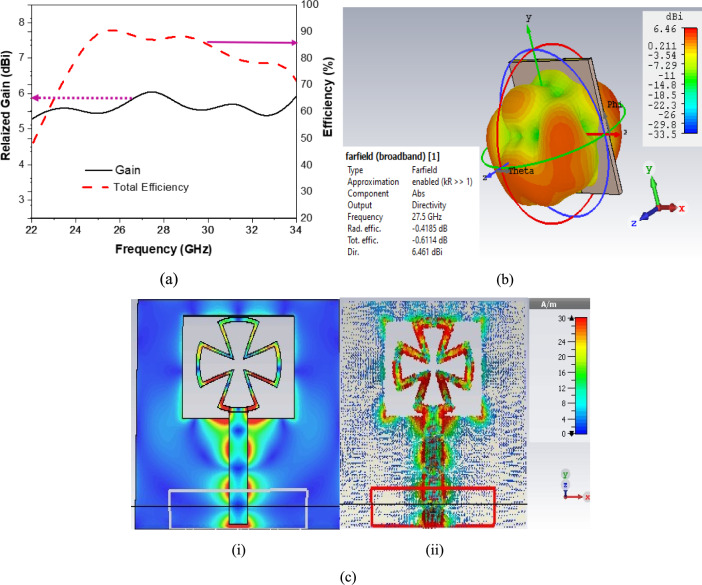
(**a**) A single antenna element response of realized gain and total efficiency, (**b**) 3D Fairfield directivity of a single antenna element, and (**c**) Current distribution of a single antenna element (i) Magnitude and (ii) Vector.

The other performance parameters of the proposed antenna are also simulated as gain and efficiency. The realized Gain of a single antenna element is shown in Fig. 3a, which has a stable gain over the operating band and obtains a maximum value of 6 dBi at 27.5 GHz. While the antenna efficiency is appropriate over the operating band, with a maximum value of 90% at 25 GHz. The 3D far-field radiation pattern is shown in Fig. 3b. This figure shows the antenna radiates most strongly in the direction of the main lobe, with a peak directivity of 6.46 dBi, which is decent for compact antennas.

The current distribution magnitude and vector over the proposed antenna are shown in Fig. 3c. This figure shows that the distribution of the current is flowing through the feedline into the slot, exciting resonant modes. High current density red regions are concentrated at the edges of the cross slot, especially near the corners and feed area. This is typical in antennas, where current tends to concentrate at sharp corners and discontinuities. Moreover, the current distribution presented in Fig. 3c(i) and (ii) provides critical insight into the resonant behavior of the antenna element. The magnitude plot confirms strong current confinement along the intended radiating edges, while the vector representation illustrates a coherent and in-phase current flow, essential for achieving the observed broadside radiation and high directivity. This alignment between current behavior, resonance frequency response, and radiation performance validates the design’s efficiency and impedance matching characteristics, underscoring the antenna’s suitability for the proposed application.

### Comparative study of two elements

**Fig. 4:**
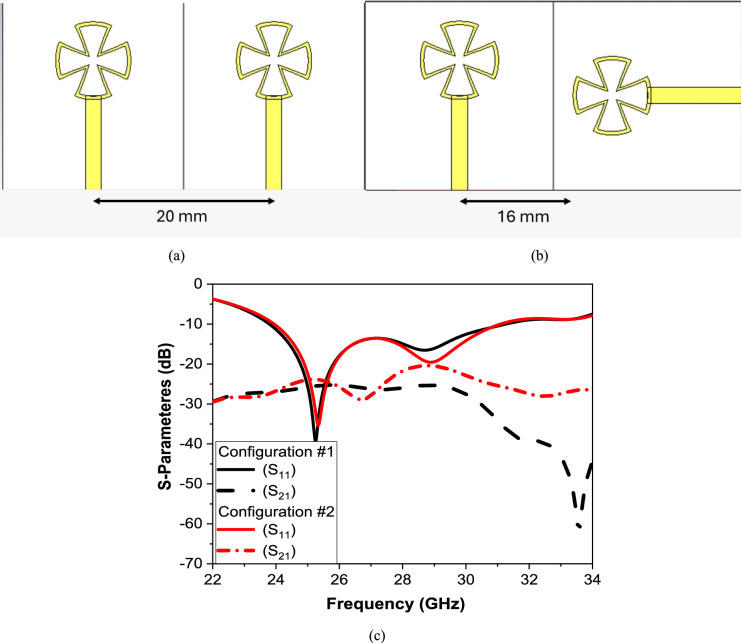
Two antenna elements parallel (**a**) Configuration #1 and (**b**) Configuration #2, and (**c**) S-parameters for two antenna element configurations.

To check the optimum distribution of antenna elements, we have studied two different configurations that could achieve miniaturization. Two parallel antenna elements have been well organized as shown in Fig. 4a Configuration (1) with separation from center to center as 20 mm. On the other hand, two perpendicular antenna elements have been well organized as shown in Fig. 4b Configuration (2) from center of vertical element to the center of horizontal element separtion almos16 mm. In addition, the other characteristic parameters, such as current distribution, gain, and efficiency, should also be studied. The result of both configurations is shown in Fig. 4c, from this figure shown that the parallel antenna elements Configuration (1) has good values for S-parameters. It dips below − 10 dB around 27 GHz, indicating good impedance matching at that frequency. Good isolation between ports, which means More power is radiated instead of being coupled between antennas.

**Fig. 5:**
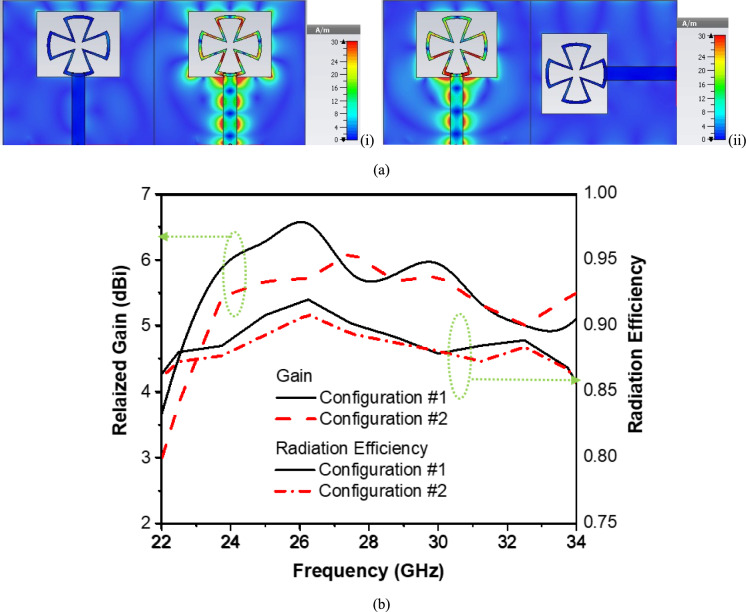
(**a**) Current distribution of two antenna elements (i) Configuration #1, (ii) Configuration #2, and (**b**) Realized Gain and Radiation Efficiency for two antenna element configurations.

The results of the current distribution for both proposed configurations are shown in Fig. 5a. It is shown that there is very good isolation between both elements, as well as configuration (1) gives better mutual coupling and isolation as the separtion between element greater than configuration (2). Moreover, the realized gain and efficiency are shown in Fig. 5b. This figure shows that both configurations maintain relatively high efficiency above 0.85 across the operating band. Configuration (1) generally exhibits slightly higher efficiency, especially in the 24–26 GHz and 30–32 GHz ranges. Configuration (1) offers a higher peak realized gain with better directivity and power delivery in some of the operating bands.

### MIMO antenna array

**Fig. 6:**
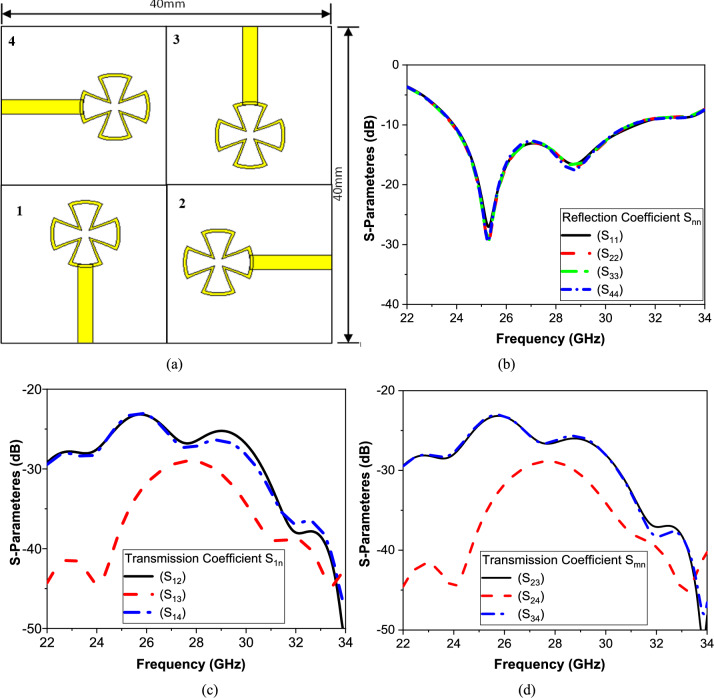
(**a**) Design model of the proposed 4 × 4 MIMO antenna, (**b**) Reflection Coefficient Snn parameters, (**c**) Transmission Coefficient S_1n_ parameters, and (**d**) Response of S_nm_ parameters.

The modified circular patch antenna was used in the design of a 4 × 4 MIMO antenna array operating at 28 GHz. The individual antennas in the array were spatially arranged orthogonally with respect to each other, as shown in Fig. 6a, to improve the isolation between the individual MIMO antennas. This configuration also has the benefit of creating circular polarization. The S-Parameters simulation results are shown in Fig. 6b–d. These results show that at frequencies 24 GHz and 32 GHz, the transmission dips are stronger, lower than − 30 dB, indicating better isolation or reduced coupling at these frequencies. The impedance matching at all four ports is better than − 14 dB, and the isolation between the radiating elements is better than 26 dB.

**Fig. 7:**
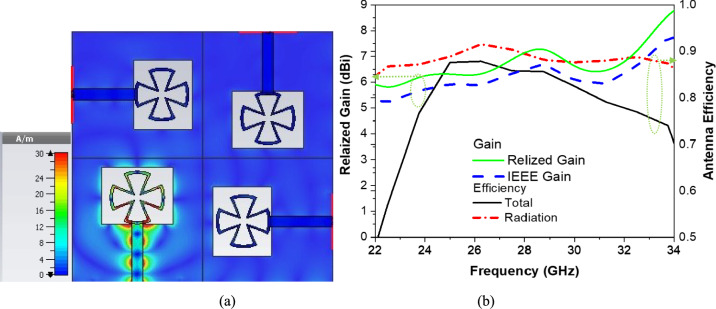
(**a**) Current distribution of proposed 4 × 4 antenna elements and (**b**) The proposed 4 × 4 MIMO antenna Gain and (**b**) Antenna Efficiency.

Figure 7a shows how the surface current is distributed across the 4 × 4 Proposed MIMO system at the resonant frequency 28 GHz. The current distribution highlights which parts of the radiating elements are most active in radiation, and the strong currents at the edges and feed points confirm effective excitation and radiation of the array. Moreover, uniform current distribution across the elements indicates good coupling and efficient array performance. Figure 7b illustrates the IEEE gain, realized gain, radiation efficiency, and total efficiency of the proposed 4 × 4 MIMO antenna over the operating frequency range. The IEEE gain represents the antenna gain assuming perfect impedance matching and accounts only for radiation characteristics, excluding mismatch losses. In contrast, the realized gain includes the effect of impedance mismatch and is defined as the product of the IEEE gain and the reflection efficiency $\left( {1\left| {S_{11} } \right|^{2} } \right)$. The radiation efficiency $\left({\eta }_{\mathrm{rad}}\right)$ quantifies the ratio of radiated power to the accepted input power and is expressed as ${\eta }_{\mathrm{rad}}={P}_{\mathrm{rad}}/{P}_{\mathrm{accepted}}$, reflecting conductor and dielectric losses. The total efficiency $\left({\eta }_{\mathrm{tot}}\right)$ accounts for both radiation and mismatch losses and is given by $\eta_{{{\mathrm{tot}}}} = \eta_{{{\mathrm{rad}}}} \left( {1 - \left| {S_{11} } \right|^{2} } \right)$. As observed in Fig. 7b, the proposed antenna maintains a high radiation efficiency exceeding approximately 85–90% across the operating band, while the total efficiency shows a slight reduction at higher frequencies due to increased mismatch and material losses. The high realized gain combined with stable efficiency confirms the suitability of the proposed 4 × 4 MIMO antenna for mm-wave 5G applications.

**Fig. 8:**
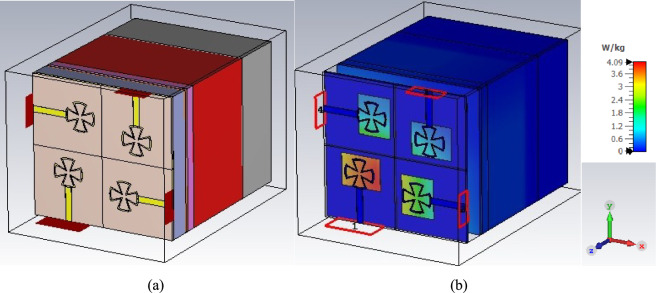
(**a**) The configuration of the MIMO system on the human hand and (**b**) SAR calculation of the proposed MIMO system.

Figure 8 shows the results of an evaluation of Specific Absorption Rate (SAR) for the phantom hand in the presence of the proposed system. SAR Performance, as shown in Fig. 8, is demonstrated using a phantom model created with dielectric materials of varying relative permittivity (ε_r_), taking into account the shape of a real human hand. A red layer representing fat with a permittivity (ε_r_) of 5 follows the top gray layer, which represents human skin with a relative permittivity of 30^29^. Blood, which has a permittivity of 60, is found beneath the fat layer, and bone, as a dark gray layer, which has a permittivity of 10, is found much deeper. The power of the IoT millimeter sensor application is 200 mW.

The SAR has been calculated at three different frequencies within the operating band at 25, 28, and 30 GHz, with SAR values at 10 g being 1.9 W/Kg, 1.626 W/Kg, and 0.8 W/Kg, respectively. For broad public exposure, IEEE limits SAR to ≤ 1.6 W/kg (averaged over 1 g of tissue), while IEC limits SAR to ≤ 2.0 W/kg (averaged over 10 g of tissue). The gadget satisfies regulatory standards for short-term exposure in controlled conditions because the average 1.426 W/kg is below both criteria. By guaranteeing that, in the worst circumstances, the tissue temperature rise stays below 1 °C, these criteria are intended to stop immediate thermal injury.

### Fabrication of the proposed MIMO system

This section examines the performance of single-element antennas and the suggested MIMO antenna system using experimental measurements and microwave electromagnetic simulations. The proposed system is fabricated by using photolithographic techniques. The S-parameter measurement is carried out using the coaxial cable to ZVA67 (Rohde & Schwarz, Columbia, MD, USA), Rohde and Schwarz vector network analyzer (VNA) from 10 MHz to 67 GHz. The fabricated prototype of the single and 4 × 4 MIMO connected to the VNA, an end-launch connector 2.4 mm from Southwest Microwave Inc. is used for this purpose.

**Fig. 9:**
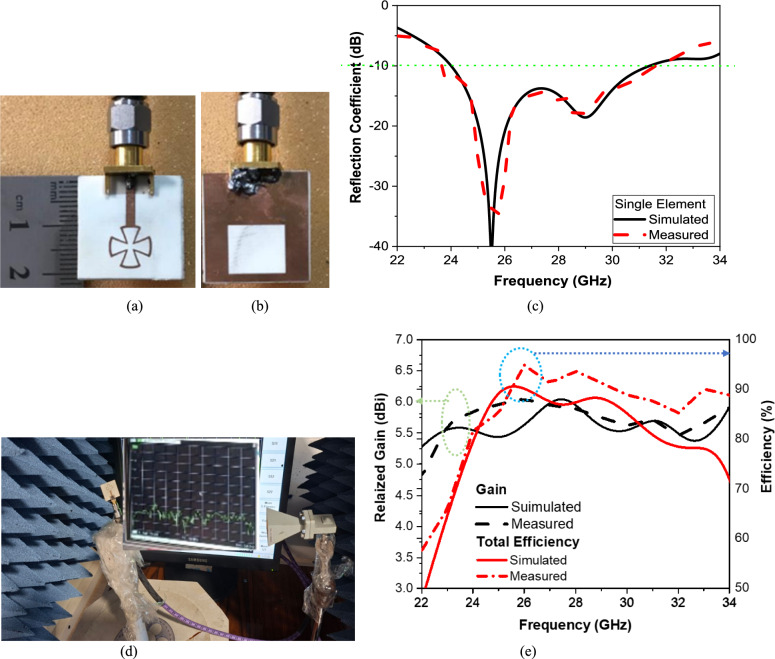
Fabrication of a single antenna element (**a**) Radiator layer, (**b**) Ground plane layer, (**c**) Simulated versus measured results, (**d**) Photo of the measurement gain setup, and (**e**) Simulated versus measured gain and efficiency of the single antenna.
- 4 × 4 MIMO element antenna performance
  The proposed MIMO system is fabricated on Rogers’ RT/Duroid 4003C substrate with a dielectric constant of 3.55, a thickness of 0.8 mm, and a loss tangent of 0.0027, as shown in Fig. 10. Figure 10a,b show the photo of the fabricated antenna upper and lower side, respectively. While Fig. 10c illustrates the gain and radiation efficiency of the proposed 4 × 4 MIMO antenna over the frequency range of 22–34 GHz, comparing simulated and measured results. The realized gain is plotted on the left axis, while the antenna efficiency is shown on the right axis. Overall, the antenna demonstrates stable broadband performance across the considered millimeter-wave band. The simulated realized gain varies approximately between 5 and 6.5 dBi, while the measured gain closely follows the same trend with slight deviations. These discrepancies are expected and can be attributed to fabrication tolerances, connector losses, substrate parameter variations, and measurement uncertainties, which are more pronounced at higher frequencies. The radiation efficiency remains consistently high throughout the band, exceeding 70% and approaching 80–85% near the upper frequency range (around 30–32 GHz). The close agreement between simulated and measured efficiency curves validates the effectiveness of the antenna design and confirms minimal conductive and dielectric losses. The slight efficiency reduction at lower frequencies can be attributed to increased substrate losses and imperfect radiation confinement in the MIMO configuration. During the measurement of S parameters, only one port receives excitation because the four antenna elements are symmetrically organized and identical, while the other ports are terminated with 50 Ω loads. Figure 10d shows simulated versus measured S-parameters of the proposed 4 × 4 MIMO antenna (i) S_11_, S_22,_ and (ii) S_33_, S_44_. These results confirm very good agreement between measured and simulated values for all ports.

- Single-element antenna performance
  The manufacture of the single antenna is shown in Fig. 9a,b. The measured and simulated S-parameters of the single antenna are shown in Fig. 9c. The measured impedance bandwidths of 8% (22–34 GHz), the antenna exhibits broadband extended from 22 to 34 GHz, as shown in Fig. 9a. The MIMO antenna array is fabricated on Roger’s RT/Duroid 4003C substrate with a dielectric constant of 3.55, a thickness of 0.8 mm, and a loss tangent of 0.0027. The overall dimension of the MIMO is 40 × 40 × 0.8 mm^3^. Figure 9d shows the mm-wave setup to measure the gain of a single antenna element. Figure 9e presents the simulated and measured gain and total radiation efficiency of a single antenna element over the frequency range of 22–34 GHz. The results demonstrate stable and broadband radiation performance, which is essential for ensuring consistent behavior when the element operates within a MIMO configuration. The total radiation efficiency exhibits a rapid increase at the lower end of the band and stabilizes at higher frequencies. The simulated efficiency exceeds 85% over a wide frequency range and reaches values close to 80–90% around 25–27 GHz, indicating minimal conductive and dielectric losses. The measured efficiency shows good agreement with the simulated results, with a small reduction at higher frequencies, likely due to additional losses introduced by the measurement setup and finite substrate conductivity. Overall, the close correspondence between simulated and measured gain and efficiency validates the accuracy of the antenna design and fabrication process. The high efficiency and stable gain of the single element ensure reliable performance when integrated into the full MIMO array, supporting the antenna’s suitability for millimeter-wave MIMO applications, such as 5G and beyond wireless systems.

**Fig. 10:**
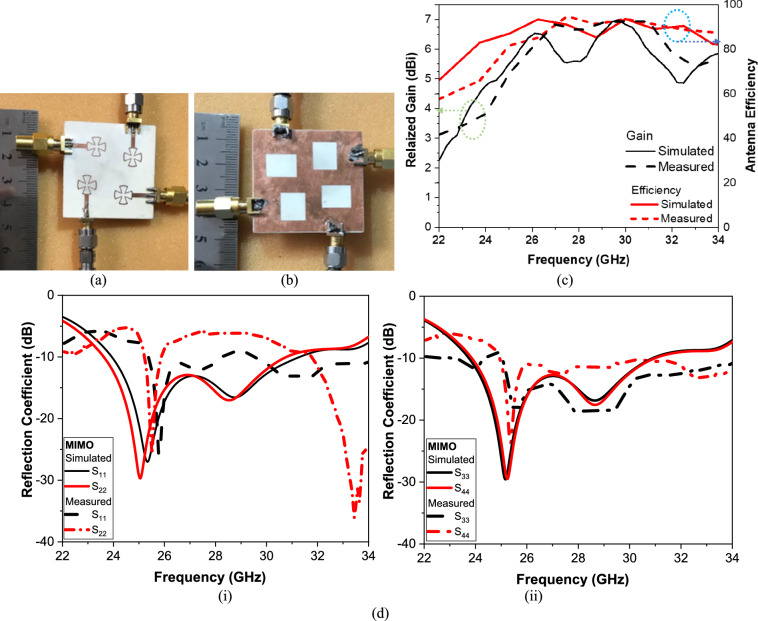
Photo of the fabrication of the proposed 4 × 4 MIMO antenna (**a**) Radiator layer, (**b**) Ground plane layer, (**c**) Simulated versus measured gain and efficiency of the proposed 4 × 4 MIMO antenna, and (**d**) Simulated versus measured S-parameters of the proposed 4 × 4 MIMO antenna (i) S11, S22, and (ii) S33, S44.
- MIMO radiation pattern antenna performance
  Through a comparative analysis that includes assessing the signals received by a standard horn antenna in an anechoic room, as shown in Fig. 11, the radiation patterns are recorded and examined. Over the operating band of 25, 28, and 30 GHz, as shown in Table 1. One antenna of the proposed MIMO system is activated during the radiation pattern measurement. The other antenna is linked with a matched load of 50 Ω with a 2.4 mm cable for the desired frequency bands, as shown in Fig. 11. This process establishes the effect of the second element on the overall gain of the single element in an array setting.

**Fig. 11:**
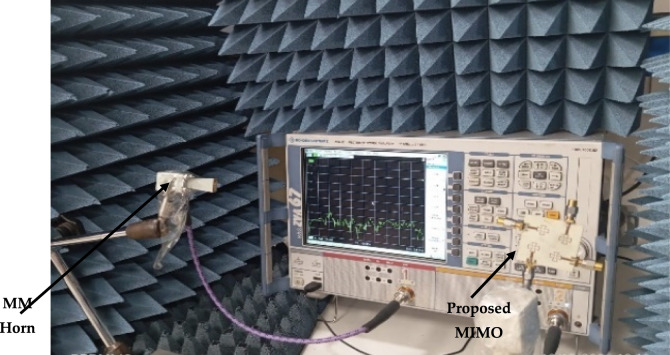
Photo of the 4 × 4 MIMO system in an anechoic chamber.

**Table 1:**
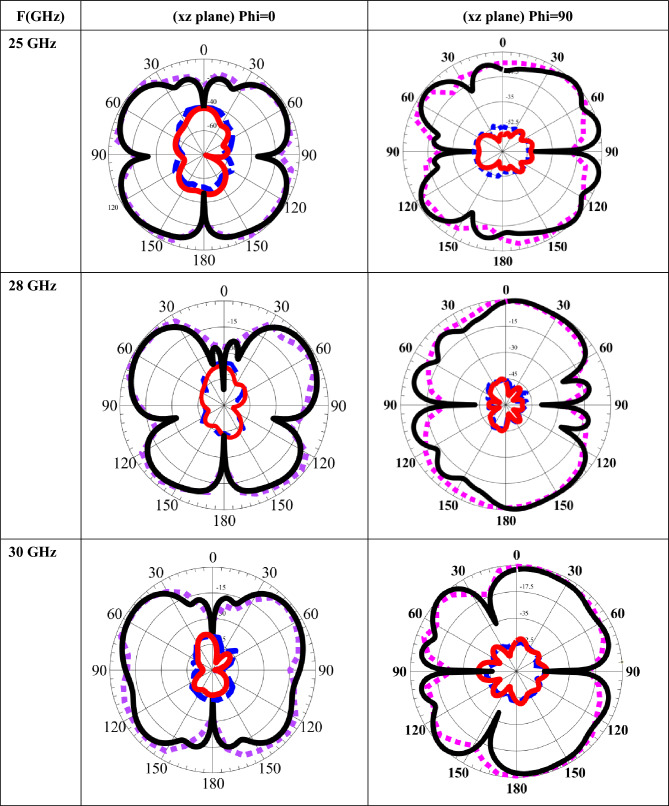
Simulated and measured radiation patterns 2-D radiation characteristics at both E & H planes for xz plane and yz plane of the proposed antenna: simulated (______ Solid line) and measured (---- Dashed line) for different frequencies.

Table 1 demonstrate a strong level of agreement for both the E-plane (xz plane, ϕ = 0°) and H-plane (yz plane, ϕ = 90°) across the operating frequencies of 25, 28, and 30 GHz. In all cases, the measured results closely follow the main-lobe directions, beamwidths, and overall radiation characteristics predicted by simulation, confirming the validity of the proposed antenna design. Minor discrepancies observed in the sidelobe levels and null depths can be attributed to practical measurement uncertainties, fabrication tolerances, connector effects, and alignment errors in the measurement setup, which are common at millimeter-wave frequencies. In this setup, a 50-Ω matched load is attached to the second port, while the first port is actively activated.

### MIMO performance parameters

To evaluate a MIMO system’s performance, the correlation characteristics between its ports must be examined. This section uses numerical simulation to evaluate the 4 × 4 MIMO system’s performance. Results are shown and discussed for the S-parameters, diversity parameters, and gain patterns. Envelope Correlation Coefficient (ECC), Diversity Gain (DG), Mean Effective Gain (MEG), Channel Capacity Loss (CCL), Total Active Reflection Coefficient (TARC), and Channel Capacity (CC) diversity performance results using a written MATLAB code^35–40^.

- ECC

ECC is among the important MIMO performance metrics associated with the correlation between two concurrently operating and closely positioned antenna elements. The parameter can be computed using far-field radiation patterns or S-parameters of the i^th^ and j^th^ antennas as mathematically given in Eqs. (3) and (4)^46,47^.

3 $$ECC = \frac{{\left| {\int {\int_{4\pi } {\left( {\vec{F}_{i} (\theta ,\varphi ) \times \vec{F}_{J}^{*} (\theta ,\varphi )} \right)d\Omega } } } \right|^{2} }}{{\int {\int_{4\pi } {\left| {\left( {\vec{F}_{i} (\theta ,\varphi )} \right)} \right|^{2} d\Omega \times \int {\int_{4\pi } {\left| {\left( {\vec{F}_{J} (\theta ,\varphi )} \right)} \right|^{2} d\Omega } } } } }}$$

where (F_i_)(θ, φ) and (F_j_) (θ, φ) are the i-th and j-th antennas’ far field radiation patterns.

4 $$ECC = \frac{{\left| {S_{ii}^{*} S_{ij} + S_{ji}^{*} S_{jj} } \right|^{2} }}{{\left( {1 - \left( {\left| {S_{ii} } \right|^{2} + \left| {S_{ji} } \right|^{2} } \right)} \right) + \left( {1 - \left( {\left| {S_{jj} } \right|^{2} + \left| {S_{ij} } \right|^{2} } \right)} \right)}}$$

where S_ii_ and S_ij_ denote the reflection and transmission coefficients.

**Fig. 12:**
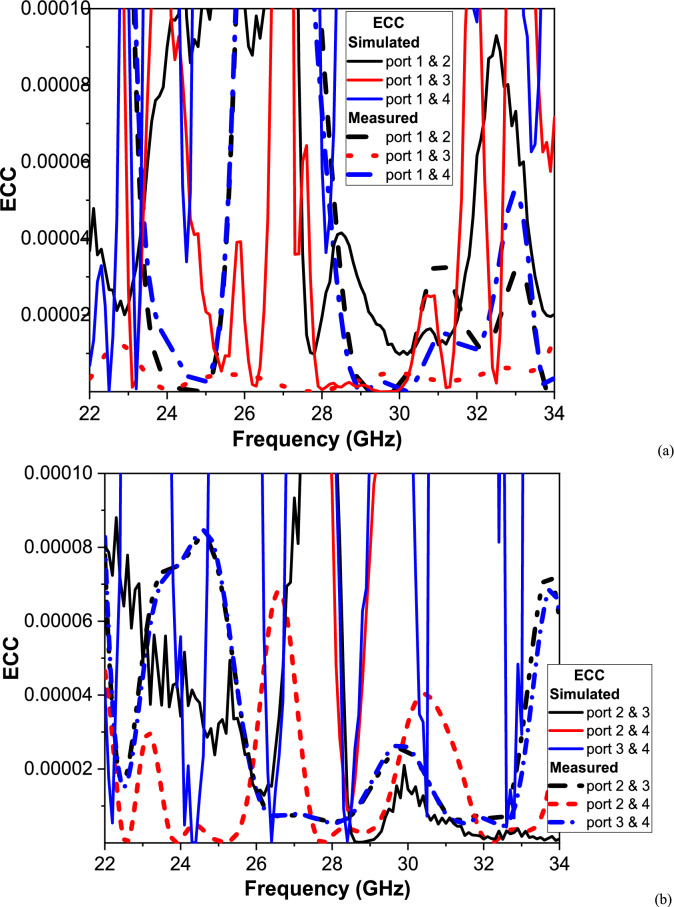
The ECC of the proposed MIMO antenna (**a**) Port 1 and (**b**) Port 2 and 3.

The Envelope Correlation Coefficient (ECC) is evaluated for all six possible antenna port pairs of the proposed 4-port MIMO system over the frequency range of 20–35 GHz. Due to the 4-port MIMO antenna design, there are six unique antenna pairs: (1, 2), (1, 3), (1, 4), (2,3), (2, 4), and (3, 4). Each pair has its own ECC values, reflecting the mutual coupling and radiation pattern correlation between the respective ports. It is concluded that all ECC values exhibit a maximum of only 0.008, which is far below the acceptable threshold (typically ECC < 0.5, and ideally < 0.1)^48^, thus demonstrating excellent diversity and isolation performance across the operational band. As illustrated in Fig. 12, the simulated ECC values (solid blue lines) remain extremely low throughout the band, with peak values well below 0.002. This confirms the superior isolation and diversity performance achieved by the proposed Greek-cross orthogonal antenna configuration, ensuring uncorrelated radiation patterns and enhanced MIMO channel capacity. The measured ECC values (red dashed lines) follow the same general trends as the simulated results, validating the antenna’s real-world effectiveness. However, minor discrepancies are observed in the form of localized peaks and fluctuations, particularly in the 24–28 GHz range, which are attributed to practical factors such as fabrication tolerances, connector losses, and measurement environment reflections. These effects are typical at millimeter-wave frequencies and do not significantly degrade overall performance.

For all port combinations, the ECC values remain exceptionally low, confirming that the antenna elements are effectively decoupled and suitable for 5G MIMO applications. Among the port pairs, the combination of Ports 1 and 2 demonstrates the closest agreement between simulation and measurement, indicating a well-balanced feed network and symmetrical antenna design. For diagonal port pairs such as Ports 1 and 4 or Ports 3 and 4, the simulation predicts nearly zero ECC, while the measurements exhibit slightly higher peaks due to asymmetrical coupling introduced during the fabrication process. Despite these practical deviations, the ECC levels are consistently minimal, ensuring reliable and efficient spatial diversity.

Overall, these results verify that the proposed 4-port MIMO antenna delivers exceptional diversity performance, with both simulated and measured ECC values indicating minimal correlation between ports. This performance makes the design a strong candidate for next-generation wireless communication systems, particularly for 5G and beyond, where high capacity, low correlation, and robust reliability are critical requirements.

(b) DG

DG is another parameter that defines the reduction in transmission power or signal-to-noise ratio improvement due to the diversity system. Mathematically, DG is computed from ECC values using Eq. (5)^49^, where a lower ECC corresponds to a higher Diversity Gain.

5 $$DG=10\sqrt{1-{\left(ECC\right)}^{2}}$$

**Fig. 13:**
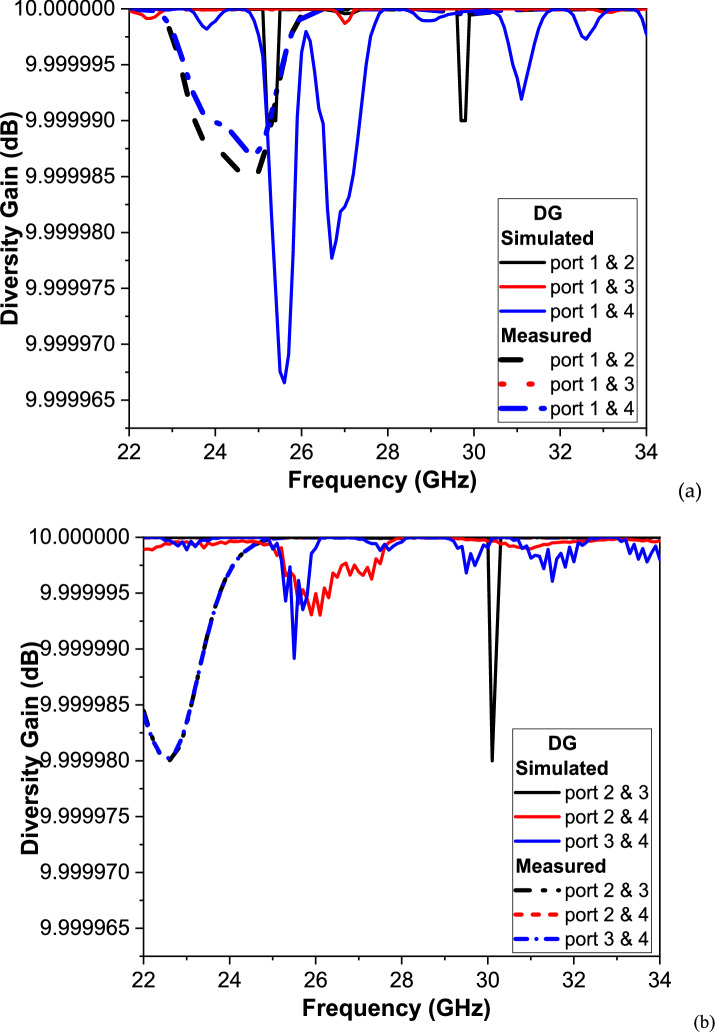
The DG of the proposed MIMO antenna.

Figure 13 presents the simulated and measured Diversity Gain (DG) of the proposed 4-port MIMO antenna across the frequency range of 20–35 GHz, covering the higher mm-Wave 5G bands. Due to the 4-port configuration, there are six unique antenna pairs: (1, 2), (1, 3), (1, 4), (2, 3), (2, 4), and (3, 4), each exhibiting distinct DG characteristics that reflect the spatial diversity performance of the system.

The simulated DG curves remain consistently close to the theoretical maximum value of 10 dB, with very minor variations across the entire band. This behavior indicates extremely low envelope correlation and confirms the strong pattern diversity and low mutual coupling achieved by the orthogonal Greek-cross antenna geometry. The maximum deviation observed in simulation is negligible, typically within ± 0.00001 dB, which demonstrates the robustness of the design for high-performance MIMO operation.

The measured DG curves (dashed red lines) follow the same general trends, validating the proposed design under real-world conditions. Slight discrepancies between simulated and measured results are observed, particularly for diagonal port pairs such as (1, 4) and (2, 4), where the measured DG shows small, localized dips near 24–28 GHz. These differences are attributed to fabrication tolerances, connector losses, and measurement setup imperfections, which are more pronounced at mm-Wave frequencies. Additionally, reflections and multipath effects in the measurement environment may have contributed to these minor fluctuations.

Importantly, for all antenna pairs, the DG values remain extremely close to 10 dB, well above the minimum requirement of 9 dB for practical MIMO systems. This confirms that the proposed design provides excellent diversity performance, with highly uncorrelated radiation characteristics and reliable spatial multiplexing capabilities. The results further highlight that the MIMO system maintains stable performance across its operational bandwidth. In particular, Port pair (1, 2) demonstrates exceptional agreement between simulation and measurement, indicating the symmetrical layout and balanced feed structure of the design. Port pairs (2, 3) and (3, 4) exhibit slightly more pronounced discrepancies, likely due to non-idealities introduced during the prototyping and measurement phases. Even at frequencies where environmental and fabrication effects are most significant, the DG never falls below 9.99998 dB, which is well within the ideal range.

Overall, these findings verify that the proposed 4-port MIMO antenna achieves outstanding diversity gain, supporting its application in 5G and beyond wireless communication systems, where high reliability, low correlation, and enhanced channel capacity are essential. The combination of simulated consistency and measured validation demonstrates the antenna’s readiness for integration into practical high-data-rate mm-Wave MIMO networks.

(c) MEG and MEG ratio

MEG is the ratio of the accepted mean power to the average incident power by the antenna in comparison to the isotropic antenna. In other words, it defines how much power an antenna receives on average under multipath propagation conditions. It is especially important for MIMO systems because it reflects signal power balance between ports and evaluates how efficiently the antenna operates in accurate environments. For a single antenna element i, MEG is calculated using the S-parameters as shown in Eq. (6)^50^ and assumes equal probability of vertically and horizontally polarized incoming signals.

6 $$MEG_{i} = 0.5\left[ {\left( {1 - \left| {S_{ii} } \right|^{2} - \mathop \sum \limits_{j = 1, j \ne i}^{N} \left| {S_{ij} } \right|^{2} } \right)} \right]$$

where S_ii_: is the Reflection coefficient at port i. S_ij: is the Transmission coefficient between port i and port j (mutual coupling). N: is the total number of antenna ports. (N = 4). For a well-designed MIMO antenna, the MEG values of all ports should be similar. To verify this balance, the MEG Ratio between two ports is calculated as shown below in Eq. (7).

7 $$MEG\, Ratio = \frac{{MEG_{1} }}{{MEG_{2} }}$$

**Fig. 14:**
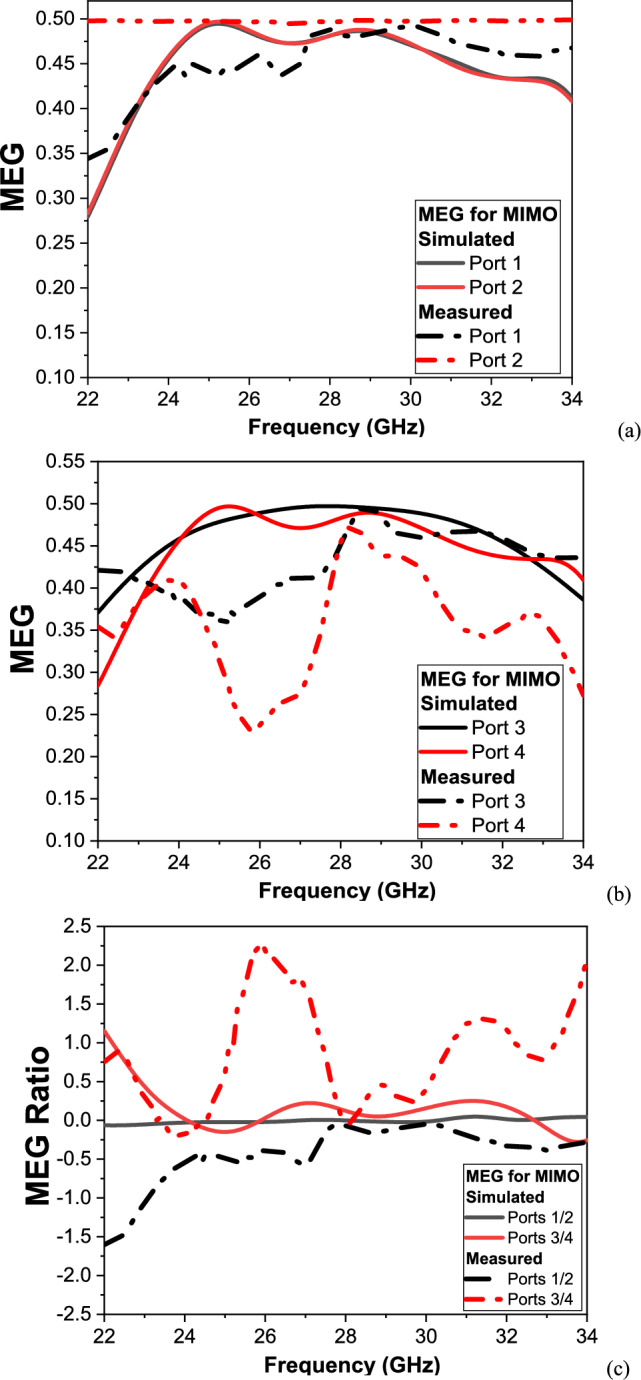
The MEG (**a**) Port 1, 2 (**b**) Port 3, 4, and MEG Ratio of the proposed MIMO antenna ports (**c**) Ports 1/2, 3/4.

Figure 14 present the simulated and measured Mean Effective Gain (MEG) and MEG Ratio of the proposed 4-port MIMO antenna over the frequency range of 20–40 GHz, targeting the higher mm-Wave 5G band (24–31.5 GHz). MEG quantifies the average received power by each antenna element in a multipath environment, while the MEG Ratio assesses the balance of received power between different ports, providing insight into the overall system symmetry and diversity performance.

The simulated MEG shown in Fig. 14a demonstrates that all four ports exhibit stable performance across the operating band. Ports 1 and 2 are nearly identical and closely approach the ideal linear value of 0.5 (− 3 dB), indicating excellent impedance matching and uniform power capture. Ports 3 and 4 also follow similar trends, with minor variations at lower frequencies, likely caused by weak edge effects and mutual coupling interactions between adjacent elements. While the simulated MEG Ratio illustrated in Fig. 14b confirms the balance of the design.

For Ports 1 and 2, the MEG Ratio remains near 0 dB across the entire band, reflecting highly symmetrical performance. Ports 3 and 4 show slightly higher fluctuations, especially below 23 GHz, yet remain well within the acceptable ± 3 dB threshold in the target 5G band. These results validate the antenna’s design for providing stable and balanced performance under ideal operating conditions. The measured MEG shown in Fig. 14a follows the general trends observed in simulation, with minor discrepancies attributed to fabrication tolerances, connector losses, and measurement setup imperfections, which are more pronounced at higher mm-Wave frequencies.

Port 2 maintains a near-ideal MEG very close to 0.5 across the entire band, demonstrating excellent real-world performance. Ports 1 and 3 show slightly lower MEG levels below 25 GHz due to increased mutual coupling and physical asymmetries in the prototype. Port 4 exhibits the most variation, particularly between 23 and 27 GHz, which may result from increased sensitivity to environmental reflections and structural inconsistencies.

The measured MEG Ratio illustrated in Fig. 14b aligns well with the simulation. Ports 1 and 2 maintain a ratio very close to 0 dB, staying well below the 3 dB limit, confirming a high degree of balance. For Ports 3 and 4, localized peaks exceeding + 3 dB occur around 26–28 GHz and near 38 GHz, likely due to coupling effects and measurement uncertainties.

However, within the primary 24–31.5 GHz 5G band, the MEG Ratio remains mostly below 3 dB, ensuring compliance with industry standards for diversity performance. Its concluded that from the simulated and measured results that the proposed 4-port MIMO antenna achieves: High MEG stability, with ports consistently delivering values close to the ideal − 3 dB, demonstrating strong multipath reception capability, Excellent port balance, especially for Ports 1 and 2, where the MEG Ratio remains consistently near 0 dB across the operating band, Acceptable variations for Ports 3 and 4, with deviations limited to narrow frequency regions outside the critical 5G band, and Strong alignment between simulation and measurement, validating the robustness of the design and confirming its suitability for high-capacity, low-correlation mm-Wave MIMO systems.

(d) CCL

The Channel Capacity Loss (CCL) is evaluated to quantify the reduction in channel capacity caused by mutual coupling and correlation between MIMO antenna elements. The procedure for calculating CCL is as follows. First, the scattering parameters of the MIMO antenna are obtained over the operating frequency band. Using these S-parameters, the normalized correlation matrix ${ alpha }^{{\mathrm{R}}}$ of the MIMO system is constructed, where the diagonal elements ${ alpha }_{{{\mathrm{ii}}}}$ represent the accepted power at the ith port and the off-diagonal elements ${ alpha }_{{{\mathrm{ij}}}}$ represent the correlation between different antenna ports. The matrix elements are calculated using Eq. (11). Subsequently, the Channel Capacity Loss is computed using Eq. (9). A lower CCL value indicates weaker correlation and better MIMO performance. In practical wireless systems, a CCL value below 0.4 bits/s/Hz is considered acceptable and indicates good diversity and channel capacity performance over the operating band^46^.

The Channel Capacity Loss (CCL) is a critical performance metric for evaluating the capacity degradation in a MIMO system due to correlation between antenna elements. It provides insight into how well the MIMO antenna can support high data rates in wireless communication systems such as 5G and beyond. The CCL quantifies the reduction in channel capacity (in bits/sec/Hz) compared to an ideal uncorrelated MIMO system. Typically, CCL < 0.4 bits/sec/Hz is considered acceptable for practical systems and excellent diversity and throughput. Where CCL < 0.4 bits/sec/Hz is considered a Poor channel performance, redesign is required. CCL provides the system channel capacity losses caused by the correlation effect of the multiple antennas. Mathematically, it can be calculated using Eq. (9) and should be below 0.4 bits/s/Hz standard limit over the working bands^46^.

9 $$C_{loss} = - \log_{2} \,det\left( {\alpha^{R} } \right)$$

where α^R^ is the normalized correlation matrix of the MIMO system which can be calculated directly from S-parameters as shown below in Eq. (10).

10 $$\alpha^{R} = \left( {\begin{array}{*{20}c} {\begin{array}{*{20}c} {\alpha_{11} } & {\quad \alpha_{12} } \\ {\alpha_{21} } & {\quad \alpha_{22} } \\ \end{array} \begin{array}{*{20}c} {\quad \alpha_{13} } & {\quad \alpha_{14} } \\ {\quad \alpha_{23} } & {\quad \alpha_{24} } \\ \end{array} } \\ {\begin{array}{*{20}c} {\alpha_{31} } & {\quad \alpha_{32} } \\ {\alpha_{41} } & {\quad \alpha_{42} } \\ \end{array} \begin{array}{*{20}c} { \quad \alpha_{33} } & {\quad \alpha_{34} } \\ {\quad \alpha_{43} } & {\quad \alpha_{44} } \\ \end{array} } \\ \end{array} } \right)$$

The accepted power at port i and the correlation between different ports are α_ii_ and α_ij_ respectively, as shown in Eq. (11).

11 $$\begin{aligned} & \alpha_{ii} = 1 - \mathop \sum \limits_{j = 1}^{N} \left| {S_{ij} } \right|^{2} \\ & \alpha_{ij} = - \left( {S_{ii}^{*} S_{ij} + S_{ji}^{*} S_{ij} } \right) \\ \end{aligned}$$

**Fig. 15:**
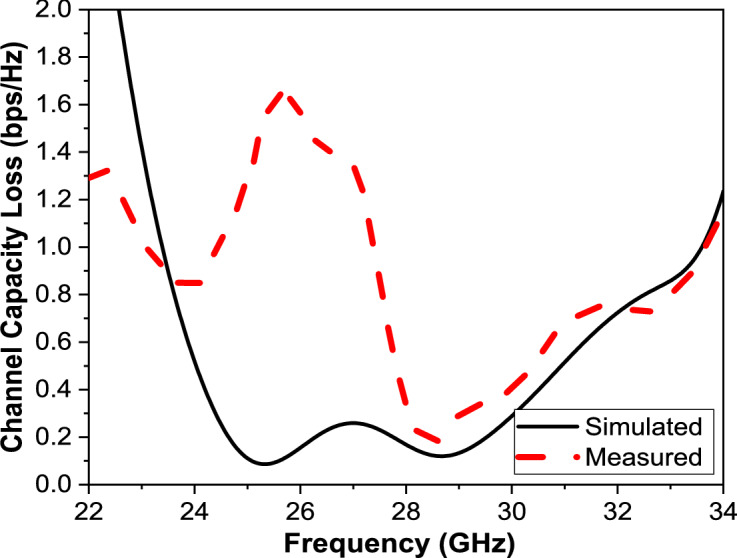
The CCL of the proposed MIMO antenna.

The Channel Capacity Loss (CCL) plot shown in Fig. 15 provides a comprehensive comparison between the simulated and measured performance of the proposed 4-port MIMO antenna system across the operational frequency band of 20–35 GHz. The blue curve represents the simulated CCL, while the red dashed curve corresponds to measured CCL values obtained from practical antenna characterization. At most frequencies, the CCL remains below the critical limit of 0.4 bits/s/Hz, which is the benchmark for high-performance MIMO systems, as we mentioned before. Specifically, in the 24.25–29.5 GHz 5G mm-Wave band, the simulated CCL approaches zero, indicating excellent channel capacity retention and minimal mutual coupling between antenna elements. The measured curve shows slightly higher values due to fabrication tolerances, connector losses, and environmental effects, yet it stays well within the acceptable range, demonstrating robust real-world performance. Maintaining a low CCL is crucial for MIMO performance, as it ensures that each antenna port contributes independently to the system’s overall capacity. The results confirm that the proposed antenna design provides: High isolation between ports, Minimal degradation of channel capacity across the operating band, and Strong suitability for 5G mm-Wave applications, particularly for dense urban environments requiring high spectral efficiency.

(e) TARC

TARC evaluates the mismatch and mutual coupling simultaneously. It can be defined as the fraction of total incident power that is reflected for a chosen excitation. It is especially important for wideband and multiport antennas. Mathematically, it can be calculated using Eq. (12)^51^. To ensure a good performance of the antenna, TARC < − 10 dB should be considered.

12 $$TARC = \sqrt {\frac{{\mathop \sum \nolimits_{i = 1}^{N} \left| {b_{i} } \right|^{2} }}{{\mathop \sum \nolimits_{i = 1}^{N} \left| {a_{i} } \right|^{2} }}}$$

where a_i and b_i are the incident and reflected waves, respectively. The four excitations (a_1_, a_2_, a_3_, a_4_) are considered in the TARC calculations, which compute the different phase combinations of the input signals. The representative phase sets for the four excitations (a_1_, a_2_, a_3_, a_4_) are (all in-phase, alternating ± , progressive, alternating progressive), respectively.

**Fig. 16:**
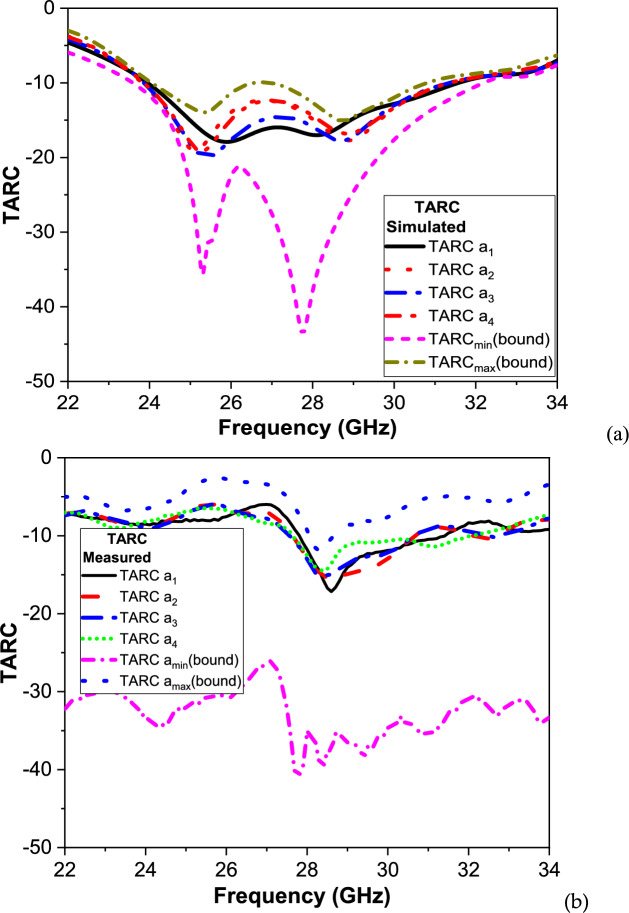
The TARC of the proposed MIMO antenna (**a**) Simulated and (**b**) Measured.

Figure 16 presents both simulated and measured Total Active Reflection Coefficient (TARC) for the proposed 4-port MIMO antenna, providing a comprehensive evaluation of the antenna’s active impedance matching and mutual coupling performance across the operational frequency band.

The simulation curve is presented in Fig. 16a. It is seen that the TARC remains well below − 10 dB across most of the 24.25–29.5 GHz 5G mm-Wave band, indicating excellent active impedance matching and minimal active reflections for multiple excitation scenarios for the four excitations (a1, a2, a3, a4) that represent different phase combinations of the input signals. For a1 (all in-phase) shows the highest TARC, representing the worst-case scenario. a3 and a4 (progressive phase excitations) provide improved matching and demonstrate the system’s ability to handle complex signal combinations. The solid black line (TARC*min*) and dashed black line (TARC*max*) represent the theoretical lower and upper bounds, respectively. All excitation curves lie between these bounds, which validates both the simulation methodology and the theoretical model. The measured curve of TARC is presented in Fig. 16b. It is seen that the TARC values follow the same general trend as the simulated curves, with the minimum TARC remaining below − 10 dB over the target band. Slight discrepancies, particularly at higher frequencies near 35–40 GHz, are attributed to fabrication tolerances in the prototype, connector losses, calibration limitations during measurement, and environmental factors such as nearby reflections during testing. The close alignment between simulation and measurement confirms the accuracy of the design process and robustness of the antenna’s real-world performance, and it maintains stable active matching even under varying phase excitations, supporting multi-stream data transmission in advanced 5G applications.

(f) CC

Channel Capacity (CC) is a key performance parameter for evaluating MIMO antenna systems that measures the loss, as it quantifies the maximum achievable data rate under given bandwidth and signal-to-noise ratio (SNR) conditions. It essentially reflects how efficiently the antenna system can handle multiple independent data streams. Mathematically, it can be calculated using the general formula or from S-parameters as shown in Eqs. (13) and (14)^52^, respectively.

13 $$CC = \log_{2} \,det\left[ {I + \frac{\rho }{{N_{t} }}HH^{H} } \right]$$

where CC is the Channel Capacity (bits per second per Hertz), I is the Identity matrix, ρ is Signal-to-Noise Ratio (SNR), N_t_ is the number of transmit antennas, H is the channel matrix representing the transmission paths between transmit and receive antennas, and H^H^ is the Hermitian transpose of H.

14 $$CC = \log_{2} \,det\left[ {I + \frac{SNR}{{N_{t} }}\left( {I - SS^{H} } \right)} \right]$$

where S is the full S-parameters of the proposed MIMO antenna.

The channel capacity of the proposed MIMO antenna is evaluated using the measured S-parameters rather than direct over-the-air measurements. The S-parameters are measured using a vector network analyzer, and the resulting data are used to compute the channel capacity based on the correlation characteristics of the antenna system, as expressed in (14). This method is widely accepted in the literature for assessing the channel capacity performance of MIMO antennas, particularly at mm-wave frequencies, where full channel sounding measurements are complex and costly*.*

**Fig. 17:**
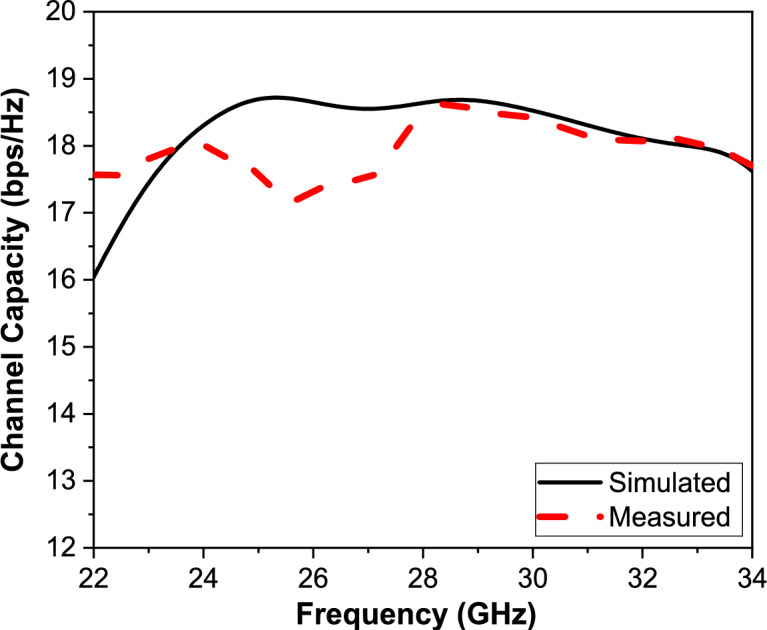
The CC simulated and measured curves of the proposed MIMO antenna.

Figure 17 illustrates the Channel Capacity (CC) of the proposed 4-port MIMO antenna system, comparing simulated and measured results across the operational frequency range of 20–35 GHz. Since the channel capacity is a critical performance metric as it represents the maximum achievable data rate per unit bandwidth under ideal propagation and noise conditions.

Figure 17 illustrates the Channel Capacity (CC) of the proposed 4-port MIMO antenna system, comparing simulated and measured results across the operational frequency range of 20–35 GHz. Since the channel capacity is a critical performance metric as it represents the maximum achievable data rate per unit bandwidth under ideal propagation and noise conditions.

The measured results (dashed red curve) follow a similar trend with slight deviations caused by fabrication tolerances, measurement environment imperfections, and connector losses. The close agreement between simulation and measurement confirms the accuracy of the design and modeling process, validating that the mutual coupling and correlation between antenna elements are well-controlled. It can be noticed that the CC remains consistently above 17 bps/Hz in most of the operational band, indicating the system’s ability to support high data rate multi-stream communication. It is concluded that the proposed MIMO antenna provides suitability for next-generation wireless systems, where maximizing channel capacity is essential to meet the demands of high-throughput applications such as 5G and beyond, and it has excellent multiplexing and diversity capabilities, ensuring reliable and efficient utilization of the available spectrum.

## Discussion

Multiple-Input High data speeds and enhanced link dependability are made possible by MIMO technology, which is a fundamental component of contemporary wireless communication systems (5G, Wi-Fi 6/7, etc.). Creating many antenna elements that are tiny, effective, and have low mutual coupling is a major difficulty for integrating MIMO systems into small devices like smartphones and Internet of Things hubs. In addition, in antenna design, co-polarization and cross-polarization components are critical factors that determine how energy is distributed across different resonance modes. While co-polarization represents the desired resonance mode, cross-polarization often stems from undesired parasitic modes or structural asymmetries. The proposed antenna design has achieved high isolation (> 22 dB) between orthogonally placed ports which is a key factor in managing polarization components for MIMO operation. To assess the performance trade-offs of thirteen distinct 4-port MIMO antenna designs, including the current work and twelve cutting-edge references, this study offers a thorough comparison analysis. The trade-offs are made across important characteristics like size, bandwidth, gain, and isolation. The analysis reveals distinct trade-offs and performance clusters among the referenced designs.

Table 2 show a comprehensive comparison between the proposed MIMO antenna and previously published MIMO antenna designs, focusing on physical characteristics, radiation performance, and MIMO system metrics. This comparison highlights the relative merits and limitations of existing approaches and clearly positions the proposed design within the current state of the art. From a size and profile perspective, the proposed antenna occupies a compact footprint of 40 × 40 mm^2^ with a substrate thickness of 0.8 mm, which is comparable to works such as^43^ and smaller than larger configurations like^42^. While some studies achieve smaller dimensions (e.g.,^38,40,53^^,^^54^), these designs often exhibit limited bandwidth, reduced efficiency, or incomplete reporting of system-level MIMO parameters. The proposed antenna offers a balanced compromise between compactness and stable electromagnetic performance, which is critical for practical wireless devices. In terms of impedance bandwidth, the proposed design achieves 8% bandwidth, outperforming several references such as^38,40,44,54–56^, where bandwidths are below 4%. Although^39,53^ report higher or multi-band bandwidths, these are often accompanied by increased design complexity or incomplete evaluation of MIMO figures of merit. The proposed antenna maintains consistent bandwidth performance while preserving compact size and low mutual coupling. Moroever, regarding gain and radiation efficiency, the proposed antenna demonstrates a moderate gain of 5.94 dBi combined with a high radiation efficiency of 93%, which is competitive with most reported designs. While higher gains are reported in^39^^,^^45^, these designs either lack efficiency data or employ larger antenna dimensions. The proposed design thus achieves an effective balance between gain enhancement and efficient radiation, which is essential for energy-efficient MIMO systems. On the other side, with respect to isolation and coupling performance, the proposed antenna achieves excellent isolation below − 25 dB, which is superior to or comparable with most references and only slightly lower than the very large antenna in^42^. This strong isolation directly contributes to improved diversity behavior. The envelope correlation coefficient (ECC) of the proposed antenna is extremely low (< 0.001), outperforming most reported works and confirming minimal correlation between antenna elements. Correspondingly, the diversity gain (DG) approaches the ideal value of ~ 10 dB, indicating excellent diversity performance.

**Table 2:** Comparison of the proposed MIMO antenna the previous antennas.

Refs	MIMO Antenna Size dimension (mm^2^)	Ports	Thickness (mm)	BW (%)	Gain (dBi)	Radiation efficiency (%)	Isolating (dB)	ECC	DG (dB)	CCL (bits/s/Hz)	TARC (dB)	MEG (dB)	SAR
This work	40.0 × 40.0	4	0.800	8.0	5.94	93.0	< − 25	< 0.0010	~ 10.0	< 0.40	< − 10	0	Included
^38^	30.0 × 30.0	4	0.787	2.0	6.10	92.0	< − 29	< 0.1600	NA	NA	NA	− 4	NA
^39^	45.5 × 45.5	4	0.800	6.6	15.48	NA	< − 25	< 0.0001	~ 10.00	0.50	− 10	− 3	NA
^40^	30.0 × 35.0	4	0.760	4.1	8.30	80.0	< − 20	< 0.0100	> 9.96	< 0.40	NA	− 3	NA
^41^	31.9 × 37.6	4	0.508	7.3	5.76	96.5	< − 18	< 0.0040	> 9.95	0.50	− 10	− 3	NA
^42^	75.0 × 100.0	4	0.508	NA	7.60, 8.12	96.0, 95.0	< − 36	< 0.5000	~ 10.0	0.50	− 5	NA	NA
^43^	40.0 × 40.0	4	0.800	6.6	4.25	97.0	< − 27	< 0.0050	10.00	< 0.40	< − 10	− 6	NA
^44^	38.0 × 36.0	4	0.800	1.6	7.20	NA	< − 23	< 0.0020	NA	NA	NA	NA	NA
^45^	31.0 × 48.0	4	0.254	5.0	10.00	NA	< − 21	< 0.0010	NA	NA	NA	NA	NA
^53^	6.2 × 6.2	4	0.254	1.8, 13.5	8.00, 10.00	90.0	< − 25	< 0.0060	~ 10.0	< 0.40	− 10	− 3	NA
^54^	16.0 × 12.0	4	0.250	0.4, 0.7	6.00, 5.80	NA	< − 20	< 0.1000	~ 10.0	< 0.28	< − 10	− 6	0.16/0.10, 0.13/0.11
^55^	41.5 × 38.5	6	1.570	0.3	4.21	70.0	< − 20	0.0100	~ 10.0	0.10	NA	− 6	NA
^56^	14.5 × 48.5	4	0.25	0.6, 0.6, 0.8	10	82.0	< − 20	< 0.0460	~ 10.0	< 0.32	− 20	− 10	0.09, 0.5, 0.7

In addition, the proposed antenna satisfies key system-level MIMO performance metrics, including channel capacity loss (CCL < 0.40 bits/s/Hz), total active reflection coefficient (TARC < − 10 dB), and balanced mean effective gain (MEG ≈ 0 dB). These parameters are either not reported or partially evaluated in many of the referenced works, limiting the completeness of their system-level assessment. The comprehensive evaluation presented in this work demonstrates the robustness and reliability of the proposed antenna in realistic MIMO operating conditions. Finally, the proposed design includes specific absorption rate (SAR) calculation, which is missing in most of the compared studies. This inclusion confirms compliance with safety standards and enhances the suitability of the antenna for portable and user-centric wireless applications. Only a few references report SAR values, and those that do often show higher values or limited frequency coverage. Overall, the comparative analysis confirms that the proposed MIMO antenna offers a well-balanced, thoroughly validated, and application-oriented solution, achieving competitive or superior performance across most critical parameters when compared to existing designs. The combination of compact size, wide bandwidth, high efficiency, strong isolation, excellent diversity characteristics, and complete MIMO metric evaluation distinguishes the proposed antenna from previously reported works. The proposed design successfully carves out a balanced and practical niche. Its key strengths are excellent wideband performance (8.0 GHz), robust MIMO characteristics, and comprehensive safety analysis with a reasonable footprint. Its core strength lies in achieving an optimal balance between wide impedance bandwidth, high isolation, and compactness using an inherently decoupled structural geometry—eliminating the need for lossy or complex external decoupling elements. The design delivers near-ideal diversity performance and stable radiation characteristics across the entire 5G mm-Wave band, all while maintaining high radiation efficiency and a single-layer, fabrication-friendly architecture. Furthermore, the modular and orthogonal arrangement ensures straightforward scalability for larger MIMO configurations. Together, these attributes position the antenna as a robust, practical, and high-performance solution for next generation 5G and beyond mobile devices, where performance metrics, integration complexity, and form factors are equally critical.

## Conclusions

Orthogonally arranged Greek cross-shaped monopole radiators. The proposed design achieves a wide impedance bandwidth of 24–32 GHz (covering key global 5G mm-Wave bands) with port isolation exceeding 22 dB, ensuring robust MIMO operation. Both simulated and measured results demonstrate excellent agreement, with stable bidirectional radiation patterns and a peak gain of 5–7 dBi across the band. The antenna exhibits high radiation efficiency (~ 93%), validating its suitability for energy-efficient 5G systems. Critical diversity metrics—including an envelope correlation coefficient (ECC) < 0.001, diversity gain (DG) > 10 dB, and channel capacity loss (CCL) < 0.001—confirm superior MIMO performance with minimal signal degradation. The Greek cross geometry, combined with strategic orthogonal placement, effectively mitigates mutual coupling while maintaining a compact footprint. These results highlight the antenna’s potential for integration into 5G devices requiring high data rates, low latency, and reliable connectivity. Future work could explore scalability for larger MIMO arrays or adaptive beamforming techniques to further enhance performance in dynamic environments.

## Data Availability

The data that support the findings of this study are available on request from the corresponding author, Dalia Elsheakh. The data are not publicly available due to privacy or ethical restrictions.
